# Changes of Date of the Atlanta Meeting of the Association of Military Surgeons

**Published:** 1908-08

**Authors:** 


### CHANGES OF DATE OF THE ATLANTA MEETING OF THE ASSOCIATION OF MILITARY SURGEONS.

The Seventeenth Annual Meeting of the Association of Military Surgeons will be held in Atlanta, October 13, 14, 15 and 16, instead of one week earlier, as was previously announced. As judging from the present outlook, this meeting promises to be one of the most successful of this distinguished Association.

According to an announcement in *The Military Surgeon*, an ably edited journal devoted to military affairs, delegates will be present from Portugal, Ecuador, Turkey, Mexico and England.

We have no hesitation in predicting that Atlanta and her physicians will extend to the military surgeons a most cordial reception.

